# mSphere of Influence: the Importance of Metabolism for Pathogen Adaptation to Host-Imposed Stresses

**DOI:** 10.1128/mSphere.00566-19

**Published:** 2019-08-21

**Authors:** Jorge Amich

**Affiliations:** a Manchester Fungal Infection Group (MFIG), Division of Infection, Immunity and Respiratory Medicine, School of Biological Sciences, Faculty of Biology, Medicine and Health, University of Manchester, Manchester Academic Health Science Centre, University of Manchester, Manchester, United Kingdom

**Keywords:** fungal virulence, *in vivo* transcriptomics

## Abstract

Jorge Amich studies several aspects of sulfur and nitrogen metabolism in Aspergillus fumigatus, with the ultimate aim of identifying targets for the development of novel antifungals. In this mSphere of Influence article, he reflects on how “Sub-Telomere Directed Gene Expression during Initiation of Invasive Aspergillosis” (A. McDonagh, N. D. Fedorova, J. Crabtree, Y. Yu, S. Kim, et al., PLoS Pathog 4:e1000154, 2008, https://doi.org/10.1371/journal.ppat.1000154) impacted his thinking about *in vivo* metabolism and how to investigate it.

## COMMENTARY

It was toward the end of my Ph.D. research when “Sub-Telomere Directed Gene Expression during Initiation of Invasive Aspergillosis” (1) was published. This article constituted the first global transcriptional signature of a fungal pathogen invading mammalian tissues. The experimental approach of this study was novel and masterful, thanks to which robust results with huge relevance to the field were reported. The authors first performed a histological time course analysis of infected murine lungs to determine the dynamics of spore germination and hyphal development in tissue. Then they optimized and validated a linear amplification method to maintain mRNA ratios, which was the bottleneck preventing global transcriptomic analyses, as the amount of fungal RNA that can be recovered from the infected mammalian tissues is insufficient for global transcriptomics. With that, they were able to compare the transcriptomes of developmentally matched Aspergillus fumigatus germlings during growth in murine lungs under a variety of *in vitro* conditions.

This article was a milestone in the field, as it provided the most complete and reliable information to date about the metabolic status and nutritional needs during infection of Aspergillus fumigatus (and any other fungal pathogen). The authors very smartly compared the organism’s transcriptomes during *in vivo* growth not only with respect to a rich medium (which uncovered and confirmed general trends of nutrient adaptation, such as adaptation to iron or zinc limitation) but also with respect to specific conditions that were suspected of being relevant for infection. For example, they compared the transcriptomes of germlings growing in the presence of hydrogen peroxide or neutrophils with those in the *in vivo* situation, and they observed that some genes follow similar trends and therefore could make partial correlations. By these means, they could establish several host-imposed conditions and found key genes for the related fungal adaptation. Not satisfied with that, the authors went even further and mapped the locations of the differentially expressed genes in the genome, which led them to identify several clusters of genes, many of which corresponded to secondary metabolites. This was among the first studies that directly demonstrated the importance of secondary metabolism for fungal virulence.

This study has been deeply influential in the way I think about the relevance of metabolism for pathogenicity, and it opened my eyes to the importance of controlling the *in vitro* conditions to mimic the *in vivo* environment as closely as possible. At the time, I was investigating A. fumigatus zinc homeostasis at neutral pH and its relevance to virulence (2, 3). I was therefore aware of the importance of zinc acquisition and pH regulation, and it was very pleasant to see that this article had detected genes related to both processes upregulated under the *in vivo* condition. In fact, this paper provides information about the potential relevance of all genes and the processes in which they are involved, so I have gone countless times, and still do, to the original tables of differentially expressed genes to check whether or not particular gene products are likely to be relevant for infection. Even more importantly, this article broadened my view of the complexity and great importance of *in vivo* metabolism and influenced me to forge my belief that metabolic versatility is an essential trait of A. fumigatus’ virulence, the unravelling of which will lead to the development of novel therapies. Based on such a conviction, my current research deals with various aspects of sulfur and nitrogen metabolism, and when I design *in vitro* assays, I always consider very carefully the experimental conditions so that the results obtained are meaningful and can be extrapolated to the *in vivo* situation.
